# Analysis of Career-Advancement for Medical School Graduates During the COVID-19 Pandemic at a Chinese Teaching Hospital

**DOI:** 10.3389/fnint.2021.739893

**Published:** 2021-12-17

**Authors:** Xiaoyan Zhu, Mingxuan Xie, Xiaobo Xia, Xiangping Li, Le Zhang

**Affiliations:** ^1^Department of Medical Graduates, Xiangya Hospital, Central South University, Changsha, China; ^2^Department of Geriatrics, Xiangya Hospital, Central South University, Changsha, China; ^3^National Clinical Research Center for Geriatric Disorders, Xiangya Hospital, Central South University, Changsha, China; ^4^Department of Ophthalmology, Xiangya Hospital, Central South University, Changsha, China; ^5^Department of Pharmacy, Xiangya Hospital, Central South University, Changsha, China

**Keywords:** COVID-19, employment, medical school graduates, pandemic, self-efficacy, career-advancement

## Abstract

The COVID-19 pandemic has led to widespread social and economic disruptions in the balance of labor market. Our study aims to analyze the career-advancement of medical school graduates during the COVID-19 pandemic and the associated influencing factors. We collected and compared the career-advancement data of medical school graduates at a Chinese teaching hospital from 2016 to 2020. A self-designed 20-element medical graduates employment questionnaire and a Chinese adaptation of the General self-efficacy scale were distributed by the Questionnaire Star platform. Univariate analysis (Pearson's Chi-square-test and Fisher's exact-test) and subsequent binary logistic regression were used. Findings demonstrated that the career-advancement rate of medical graduate students in 2020 is 71.3%, which is significantly lower than that for the preceding 4 years from 2016 to 2019 (*p* < 0.001). Of the 251 employed medical school graduates, 159 (63.3%) have signed an employment agreement or contract, 83 (33.1%) are pursuing continued education domestically, and 9 (3.6%) have offers from foreign institutions. Univariate analysis revealed statistical differences of medical graduates' employment among various specialties, oral defense completion, job search start date, CV submission times, participation in a probationary period, and self-efficacy. Significant predictors for successful employment were early job search and self-efficacy by logistic regression model (χ^2^ = 12.719, *p* < 0.001). Most medical graduates assumed that the COVID-19 pandemic had a major (40.6%) or moderate (48%) impact on career-advancement. The COVID-19 pandemic has profoundly impacted the career-advancement of medical school graduates in 2020. We should make adaptive changes to improve the career-advancement of medical graduates.

## Introduction

Every year January to March is the traditional job-hunting season for university students. Unfortunately, this period coincided with the emerging coronavirus disease 2019 (COVID-19) pandemic caused by the severe acute respiratory syndrome coronavirus 2 (SARS-CoV-2) in early 2020. As of October 27, 2021, the pandemic has caused more than 244 million confirmed cases and 4.9 million deaths worldwide reported by WHO (Xie and Chen, 2020; WHO, 2021). COVID-19 is much more than a health crisis, but also has profound and long-lasting economic and psychological consequences that endangers community health. People are experiencing especially elevated levels of stress and depression. Such mental health effects stemming from the COVID-19 pandemic, like the virus itself, are well-documented not only in college students, but also among healthcare workers (Sahin et al., 2020). It has also triggered one of the worst job crisis since the Great Depression in 2007–2008. Such consequences are the result of countermeasures to curb the transmission of the virus, including social distancing, lockdowns, and world-wide reductions in production and consumption. The estimated overall unemployment rate in the United States nearly quadrupled between February and May of 2020 and soared to 14.7% in April (Washington Post, 2020). A global multiregional macroeconomic model was developed and it predicted the global consumption decline amount to $3.8 trillion, triggering significant job (147 million full-time equivalents) and income ($2.1 trillion) losses (Lenzen et al., 2020). Asia, Europe, and the United States have been the most heavily impacted regions.

Large amounts of layoffs and factory closure brought by the pandemic has resulted in an economic shock worldwide. In terms of different occupation, the tertiary industry sectors, including retail, transport, tourism, and entertainment have been affected the most. As for healthcare providers, most attention has been appropriately focused on the access and delivery of care to those who are infected by SARS-CoV-2 or vulnerable to the virus. But what about employment of medical students and healthcare workforce? Some people predicted the pandemic would boost medical school graduates' employment because of the increasing demand for healthcare workers by the overwhelming surge in COVID-19 infected patients, as well as the invulnerable nature of the occupation. On the other hand, some people expected the workforce demand for medical school graduates to decline, given the negative effect of the pandemic on the overall economic situation and the adaptation to reallocation of clinical care delivery and medical resources to preserve a service during the pandemic. A recent survey of physician respondents in the USA reported that 97% of medical practices have been negatively affected directly or indirectly by COVID-19, with a 70% decrease in elective surgical procedures and a 33% decline in office visit of clinics, to provide critical equipment, space, and staff for critically ill medical patients with COVID-19 (Satiani et al., 2020). Another massive survey of general practice respondents in Australia reported that the COVID-19 pandemic substantially influenced their regular practice by decreased bookings (73%) and practice income (77%), with increased overall workload (61% of respondents), practice costs (81%), phone calls (93%), non-clinical staff time (76%) and non-billable time and activity (74%) (Kippen et al., 2020). A study by Satiani and Davis (2020) reported that employed surgeons were being furloughed, terminated, or persuaded to agree to a significant cut in pay, forego bonuses, or take leave without pay as healthcare systems and some physician groups started to feel the consequences of halting elective procedures. What's more, newly hired surgeons were forced in a few cases to agree to delays in starting their job, new amendments, changes in employment status, and other terms for fear of losing their job. However, no studies have been released that analyzed the employment data of medical school graduates in the context of the COVID-19 pandemic so far.

In this study, we first investigated and analyzed the career-advancement status and associated influencing factors of medical graduates at a Chinese comprehensive hospital. Based on these results, we attempted to provide a glimpse of career-advancement landscape of medical graduates in China and worldwide during the COVID-19 pandemic, and appeal to education administrators, universities, and graduates themselves to make suitable transformations in vocational planning.

## Materials and Methods

### Participants and Sampling

The career-advancement data of medical school graduates at Xiangya Hospital, Central South University, which is a comprehensive tertiary hospital in Central China, and one of the nine members in China Consortium of Elite Teaching Hospitals, were collected from 2016 through 2020 from the official statistics platform. An anonymous self-designed 20-element medical graduates employment questionnaire (MGEQ) and a Chinese adaptation of the General self-efficacy scale (GSES) (Cheung and Sun, 1999) were applied to seek out employment associated factors of medical graduates. The self-designed MGEQ was divided into three parts, namely: sociodemographic characteristics (gender, marital status, specialty), career-advancement information (career-advancement status, form of employment, type of employer, regional distribution, employee priority, method of job search, start date of job search, salary requirement, probationary period, and so on), and subjective opinion (impact of COVID-19, online recruitment efficiency, job satisfaction, career-advancement associated factors, and improvement measures and so on). The Chinese adaptation of the GSES has 10 of the original items. Each item can be answered using a 4-point Likert Scale ranging from 1 (not at all true) to 4 (exactly true). The scores on this scale range from 10 to 40, taking 20 and 30 as cut-off values for low, medium, and high self-efficacy, where higher scores indicate stronger general self-efficacy.

### Procedures and Measurements

We collected the career-advancement data of medical school graduates at Xiangya Hospital, distributed two questionnaires (MGEQ and GSES) *via* the Questionnaire Star platform. Answers were collected in a Microsoft Excel database and analyzed in SPSS (described below).

Career-advancement rate was calculated as the number of employed students divided by the number of students expected to graduate. Students were defined as career-advanced if they had a definite assignment after graduation, including a job offer at a hospital or company, plans for continued education (including doctoral studies, standard residency programs or senior specialist training), or further education at foreign institutions. Pertaining to regional distributions, our nation is divided into four economic regions according to National Bureau of Statistics of China. They are the eastern region, western region, central region, and northwest region (National Bureau of Statistics of China, 2015). The eastern region represents the economically well-developed regions in China, whereas the western region represented the economically underdeveloped regions in China. Our institution is located in the central economic region. Probationary period is the time in which the employee's practice in the desired institution is observed before formal employment. During this observation period, medical graduate students will gain precious working experience and tangible understanding of the desired hospital or institution. A successful employment depends on the satisfaction of employee's performance, salary, and working conditions of both sides.

### Statistical Analysis

We performed univariate analysis by Pearson's Chi-square test to compare the differences of career-advancement rate from 2016 through 2020, and among each individual associated factor. When the expected frequencies are ≤ 5 in one or more cells, Fisher's Exact-test was applied. These variables included gender, marital status, specialty, employee priority, geographic priority, salary requirement, method of job search, completion of oral defense, start date of job search, curriculum vitae (CV) submission times, and participation in a probationary period. We included significant variables from the univariate analysis into the binary logistic regression with a 95%CI to determine significant employment-associated factors among students who had signed an employment agreement or contract. We excluded students pursuing continued education or going abroad, so as not to confound the data with responses from students who had not undergone a job search. We performed all statistical tests using SPSS (Version 23.0). *p*-Value < 0.05 was considered significant.

## Results

### Sociodemographic Features

A total of 352 medical school graduates out of 422 (response rate 83.4%) responded to our questionnaire, which was voluntary. Of these respondents, 150 were men and 202 were women, 79 were married and 273 were single. For medical specialty distribution, there were 81, 83, 8, 9, 5, 3, 46, and 117 students in internal medicine, surgery, OB/GYN, pediatrics, intensive care, infectious disease, auxiliary specialties (mainly ultrasonic, laboratory medicine, radiology and pharmacy) and other specialties (anesthesiology, neurology, dentistry, and geriatrics).

### Change of the Career-Advancement Rate From 2016 to 2020

**Figure 1:**
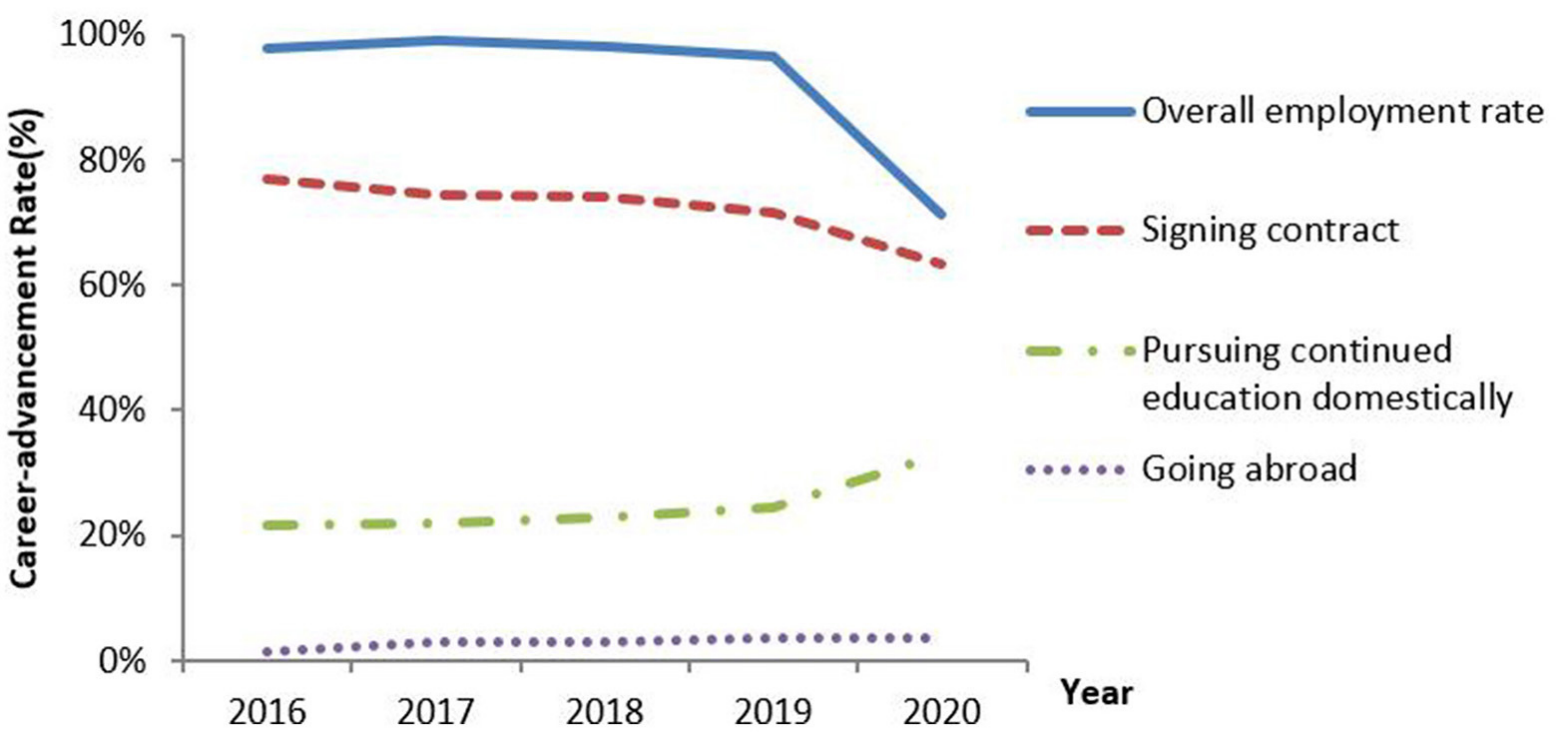
Declined career-advancement rate of medical school graduates in 2020 compared with the preceding 4 years.

As shown in Figure 1, the career-advancement rate of medical school graduates in our institution was 71.3% by July 20, 2020, which was much lower than the career-advancement rate from 2016 to 2019, ranging from 96.5 to 99.2%, with an average of 97.9%. Out of 251 employed medical school graduates, 159 (63.3%) had- signed an employment agreement or contract, which declined from its highest, which was 77% in 2016 within the past 5 years; 83 (33.1%) are pursing continued education, increased from its lowest level of 21.8% to the highest 33.1% in 2020; and 9 (3.6%) have offers from foreign institutions, a similar rate in past 5 years (Table 1). Medical students who are unemployed may pursue continued education. The regional distribution of employment represented the ability of different economic regions to absorb the medical school graduates. The eastern region represents the economically well-developed regions in China, where there are more employment opportunities. In 2020, the employment rate of our medical graduates who went to the eastern region reached the lowest level. The western region represented the economically underdeveloped regions in China, where shrinking employment opportunities were more obvious. More students preferred to seek stability and chose to work in their hometowns, who in central China were complying with the lockdown policy. The portion of students who went back to their hometown remarkably increased to 84.9% in 2020 compared with the preceding 4 years (*p* < 0.001). The type of employer was restricted to hospitals and companies in 2020 (Table 1).

**Table 1:** Career-advancement of medical school graduates from 2016 through 2020.

	**2016**	**2017**	**2018**	**2019**	**2020**	***P*** **-value**
**Overall career-advancement**						<0.001
Career-advanced	317 (97.8%)	350 (99.2%)	377 (98.2%)	361 (96.5%)	251 (71.3%)	
Career-undefined	7 (2.2%)	3 (0.8%)	7 (1.8%)	13 (3.5%)	101 (28.7%)	
**Form of career-advancement**						0.021*
Signing employment contract	244 (77.0%)	262 (74.9%)	280 (74.3%)	259 (71.7%)	159 (63.3%)	
Pursuing continued education	69 (21.8%)	77 (22.0%)	86 (22.8%)	89 (24.7%)	83 (33.1%)	
Offers from foreign institutions	4 (1.2%)	11 (3.1%)	11 (2.9%)	13 (3.6%)	9 (3.6%)	
**Type of employer**						0.002*
Hospital	238 (97.6%)	246 (94.3%)	270 (96.4%)	229 (88.5%)	149 (93.7%)	
Academic	3 (1.2%)	3 (1.1%)	5 (1.8%)	4 (1.5%)	0	
Company	3 (1.2%)	10 (3.8%)	4 (1.4%)	21 (8.1%)	10 (6.3%)	
Others	0	2 (0.8%)	1 (0.4%)	5 (1.9%)	0	
**Regional distribution**						<0.001*
Eastern region	24 (9.8%)	2 (0.8%)	218 (89.3%)	24 (9.8%)	2 (0.8%)	
Western region	26 (10.0%)	5 (1.9%)	230 (88.1%)	26 (10.0%)	5 (1.9%)	
Central region and others	52 (18.6%)	8 (2.9%)	220 (78.6%)	52 (18.6%)	8 (2.9%)	
**Hometown**						<0.001*
Yes	90 (36.9%)	105 (40.2%)	75 (26.8%)	107 (41.3%)	135 (84.9%)	
No	154 (63.1%)	156 (59.8%)	205 (73.2%)	152 (58.7%)	24 (15.1%)	

* *p < 0.05 were considered significant*.

### Factors Associated With Employment

In our study, medical graduates who had signed or were ready to sign employment contracts were included to identify employment-associated factors (*N* = 260). As shown in Table 2, univariate analysis revealed that the employment of medical school graduates was remarkably different across various factors such as medical specialty (*p* = 0.011), oral defense completion (*p* = 0.007), start date of job search (*p* < 0.001), CV submission times (*p* < 0.001), participation in a probationary period (*p* = 0.020), and self-efficacy (*p* < 0.001). For medical specialties, students from internal medicine and infectious disease were more likely to find jobs during the pandemic. Students who have completed their oral defense, started early to search for jobs, submitted their CV many times, participated in a probationary period, and have high self-efficacy were more likely to find jobs. The correlation coefficient between these variables from the univariate analysis was < 0.7, which eliminates the problem of collinearity and was suitable for subsequent logistic regression. Logistic regression identified start date of job search and job search self-efficacy as independent predictors of employment (model χ^2^ = 12.719, *p* < 0.001, Table 3).

**Table 2:** Univariate analysis of employment-associated factors.

	**Employment**	**Unemployment**	***P*** **-value**
**Gender**			0.157
Male	61 (23.4%)	48 (18.5%)	
Female	98 (37.7%)	53 (20.4%)	
**Marital status**			0.474
Married	40 (15.4%)	30 (11.5%)	
Single	119 (45.8%)	71 (27.3%)	
**Specialty**			0.011*
Internal medicine	51 (19.6%)	13 (5%)	
Surgery	28 (10.7%)	29 (11.2%)	
Intensive care	2 (0.8%)	1 (0.4%)	
Infectious disease	3 (1.2%)	0	
Auxiliary	22 (8.4%)	13 (5%)	
Others	53 (20.4%)	45 (17.3%)	
**Employee priority**			0.379
Platform	65 (25%)	34 (13.1%)	
Salary	24 (9.2%)	21 (8.1%)	
Individual development	54 (20.7%)	31 (11.9%)	
Location	7 (2.7%)	9 (3.5%)	
Family	9 (3.5%)	6 (2.3%)	
**Method of job search**			0.696
Social recruitment	132 (50.8%)	80 (30.7%)	
Campus recruitment	16 (6.1%)	12 (4.6%)	
Tutor recommendation	8 (3.1%)	4 (1.5%)	
Others	3 (1.2%)	5 (2.0%)	
**Oral defense**			0.007*
Complete	109 (41.9%)	53 (20.4%)	
Incomplete	50 (19.2%)	48 (18.5%)	
**Job search start date**			<0.001*
Before 2020	93 (35.8%)	35 (13.5%)	
January to March, 2020	39 (15%)	8 (3.1%)	
April to June, 2020	22 (8.4%)	24 (9.2%)	
Just/not begin	5 (1.9%)	34 (13.1%)	
**Geographic priority**			0.222
Eastern region	54 (20.7%)	23 (8.8%)	
Western region	5 (1.9%)	2 (0.8%)	
Central region and others	26 (10%)	21 (8.1%)	
Hometown	74 (28.5%)	55 (21.2%)	
**Salary requirement (/month)**			0.667
3–5k	2 (0.8%)	1 (0.4%)	
5–10k	33 (12.7%)	26 (10%)	
10–15k	88 (33.9%)	57 (21.9%)	
>15k	36 (13.8%)	17 (6.5%)	
**CV submission times**			0.001*
0	0	8 (3.1%)	
1–5	105 (40.4%)	56 (21.5%)	
5–10	27 (10.4%)	23 (8.9%)	
10–15	24 (9.2%)	10 (3.8%)	
>15	3 (1.2%)	4 (1.5%)	
**Self-efficacy**			<0.001*
High	109 (41.9%)	17 (6.5%)	
Medium	5 (1.9%)	15 (5.8%)	
Low	45 (17.3%)	69 (26.6%)	
**Participation in a probationary period**			0.020*
Yes	50 (19.3%)	18 (6.9%)	
No	109 (41.9%)	83 (31.9%)	

* *P < 0.05 were considered significant*.

**Table 3:** Logistic analysis of employment associated factors.

**Variables**	***B***	**Wald χ2**	***P*** **-value**	**OR**	**OR 95% CI**	
					**Lower limit**	**Upper limit**
Job search start date (just/not yet begin as reference)		30.409	<0.001*			
Before 2020	2.872	24.895	<0.001*	17.666	5.718	54.582
January to March, 2020	3.235	22.726	<0.001*	25.417	6.721	96.123
April to June, 2020	1.971	9.845	0.002*	7.180	2.096	24.599
Self-efficacy		42.399	<0.001*			
(low self-efficacy as reference)						
High	2.169	35.942	<0.001*	8.748	4.305	17.777
Medium	−0.740	1.579	0.209	0.477	0.150	1.514

*OR, Odds Ratio; 95%CI, 95% Confidence Interval*.

* *P < 0.05 were considered significant*.

### Subjective Opinions on the Career-Advancement During the COVID-19 Pandemic

**Figure 2:**
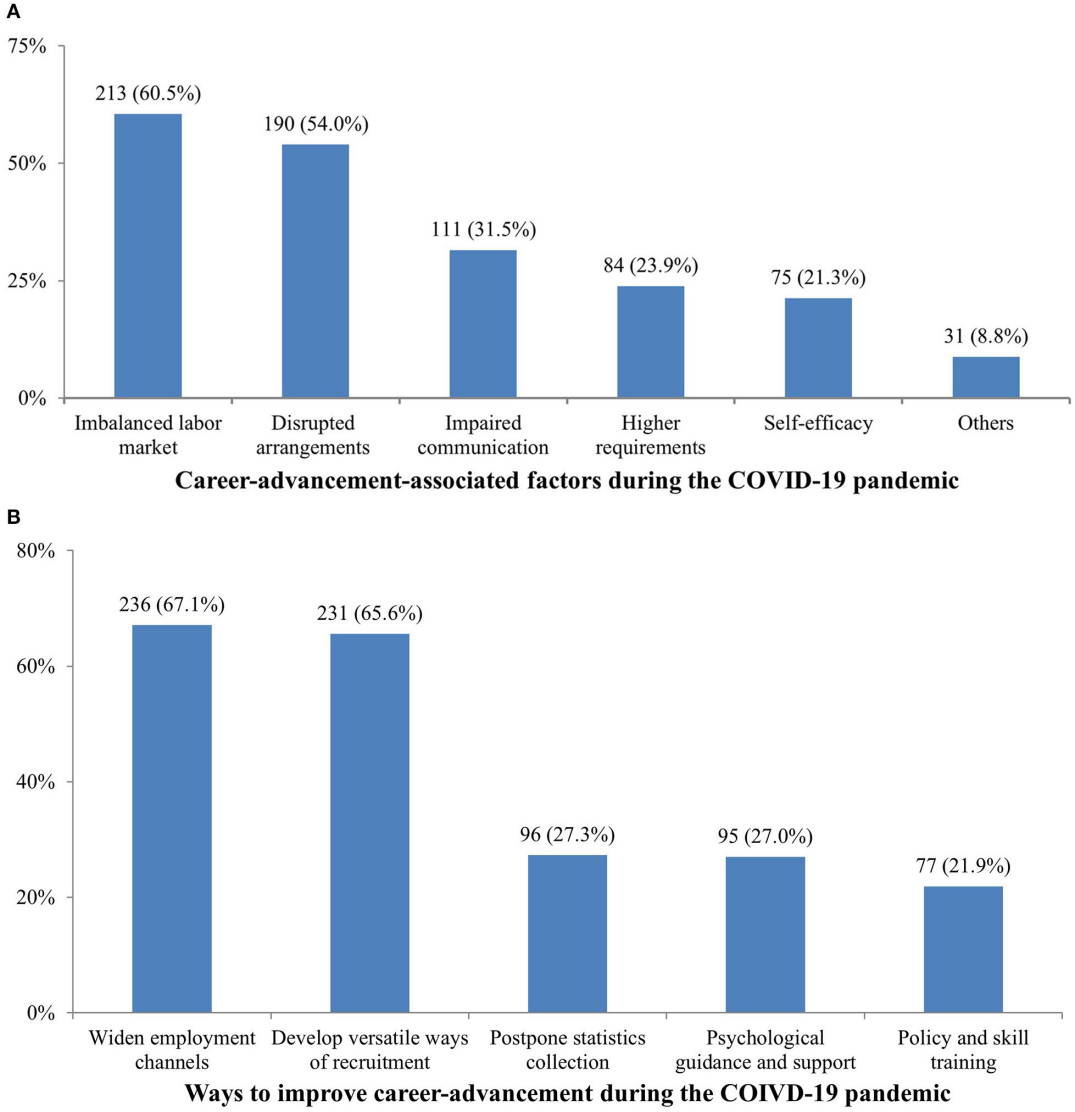
Career-advancement associated factors and potential improvement measures during the COVID-19 pandemic from a graduates' perspective. **(A)** Career-advancement associated factors during the COVID-19 pandemic from the graduates' perspective. **(B)** Career-advancement improvement measures during the COVID-19 pandemic from the graduates' perspective.

Our survey first investigated respondents' opinions about the impact of the COVID-19 pandemic on the career-advancement of medical school graduates in 2020. Most medical school graduates (88.7%) assumed that the COVID-19 pandemic had a significant negative impact on employment. Among medical school graduates, 40.6% of them assumed the influence was significant, 48.0% of them considered the impact to be moderate, and only 11.4% of them assumed it had little or no effect. Opinions on online recruitment and job satisfaction are also summarized in Table 4. The survey also demonstrated influencing factors of career-advancement during the COVID-19 pandemic from the graduates' perspective, including imbalanced labor market (reduced recruitment and increased employees), disrupted teaching and graduation arrangements, impaired communication between graduates and employers, higher requirement for sophisticated employees, and job search self-efficacy of medical graduates (Figure 2A). Among these factors, the shrinking of employment opportunities and the disruption of employment and teaching arrangements were the most important factors. Ways to improve career-advancement during the COIVD-19 pandemic were identified from our questionnaire (Figure 2B), such as to widen employment channels, develop versatile ways of recruitment, post-pone statistics collection, psychological guidance and support, policy, and skill training.

**Table 4:** Subjective opinions of medical graduates on the career-advancement during the COVID-19 pandemic.

	**Number**	**Percent**
**Impact of COVID-19**		
Major	143	40.6%
Moderate	169	48.0%
Little/no	40	11.4%
**Online recruitment efficiency**		
Effective	186	52.8%
Neutral	128	36.4%
Ineffective	38	10.8%
**Job satisfaction**		
Satisfied	175	49.7%
Neutral	82	23.3%
unsatisfied	14	4.0%
NA	81	23.0%

## Discussion

### Career-Advancement Associated Factors of Medical School Graduates

The COVID-19 pandemic destroyed the balance of demand and supply in labor market, leading to an immediate and massive reduction in labor demand, working hours, and earnings, as reported by the International Labor Organization (2020). The onset of the economic downturn resulted in a dramatic increase in the rates of youth unemployment, reflecting the difficulties and obstacles that young people face in finding jobs and getting integrated in the labor market (Nikos et al., 2020).

Hospitals, as prevention locations of the pandemic, strictly limited the volume of outpatients and inpatients, and reduced elective surgical procedures and ambulatory clinical services, in accordance with COVID-19 infection control and mitigation efforts. Consumers' health behavior, especially those with chronic conditions, changed from regular outpatient visits to skipped or post-poned elective visits, to avoid any exposure to the hospital environment to avoid possible contact with SARS-CoV-2. Additionally, the increased costs of providing personal prevention equipment, sterilizing spaces, and equipment, all resulted in decreased total income and increased hospital expenditure. It was reported that 97% of physicians were experiencing negative financial effect because of COVID-19, such as being furloughed, unemployed, persuaded to agree to a significant cut in pay, and forego bonuses (Satiani and Davis, 2020). There was also an increasing demand for proficient medical graduates in 2020, resulting in increased competition among novice medical graduates. In our survey, about 23.9% of the medical school graduates thought that employers increased the required qualifications for job applicants. The employment landscape for graduates in 2021 was more complex and challenging than in recent years.

The COVID-19 pandemic significantly impeded communication between medical graduates and employers. Both the American Association of Medical Colleges and the Ministry of Education in China established general guidelines for medical schools and suspended students' return to campus and clerkships (Whelan and Young, 2020). Hospitals and enterprises canceled large-scale campus recruitment in spring when most medical graduates were taking online courses and preparing for their job interviews online or in their hometown. In our institution, the first round of oral thesis defense and degree commencement was post-poned to July 6, the oral thesis defense rate of medical school graduates was 64.43%, far below the average rate of over 90% in the same period of recent years. Less than a quarter of medical school graduates (22.16%) participated in a probationary period, which improved the understanding of both sides and increased the possibility of being employed (73.5 vs. 56.8%). Consequently, the proportion of students pursuing continued education, rather than starting a job, was as high as 33.1%. Among medical school graduates who had signed an employment agreement or contract, 84.91% reported returning to their hometown, much higher than the 26.8–41.3% from 2016 through 2019. Additionally, the International English Language Testing System (IELTS) tests in February and March were canceled, and Proclamation 10014 issued by the U.S. government on April 22, 2020, suspended the entry of H-1B, H-2B, J, and L non-immigrant visa programs for 60 days (The Whitehouse, 2020).

Job search behaviors and outcomes were associated with psychological status, most notably self-efficacy, depression, and anxiety (Brown et al., 2006). Findings from the Penn State University's Center for Collegiate Mental Health 2019 Annual Report on Mental Health Trends Across U.S. Colleges and Universities show an increase in anxiety and depression over the last 8 years (Sahin et al., 2020). The sense of uncertainty about employment and other emotional issues, including stress, depression, anxiety, and fear were common in medical graduates. Many dimensions of interpersonal experience and embodied interactions, which are fundamental to human life, have been affected by lockdowns and other social distancing measures as well (Sahin et al., 2020). We collected our data through the Questionnaire Star platform which was not compulsory. Data were processed by EXCEL and SPSS. There were 83.4% out of 422 medical school graduates who responded to our questionnaire. In our survey, 21.3% of the medical school graduates thought the long holidays promoted inertia and negatively affected their mental health. About 46.3% were not sure whether they could successfully find jobs and 8.5% had little or no confidence. Additionally, the percentage of underemployed and unsatisfied graduates increased. Only less than half of the medical graduates reported feeling satisfied with their prospective, while 23.3% felt neutral and 4.0% were unsatisfied and hoping for a better job.

### Potential Ways to Improve Career-Advancement of Medical School Graduates During the COVID-19 Pandemic

In consideration of the employment prospects of medical graduates nationally during the pandemic, government should make prioritize increasing investment in healthcare staff and widen their ways of getting employed. Chinese government has taken immediate and effective measures to invest in the healthcare providers. On the other hand, China has successfully controlled the pandemic to a certain low level with only 125,686 confirmed cases and 5,696 deaths reported on October 27, 2021 (WHO, 2021). The medical system has gradually returned to normal with an increased volume of inpatients and outpatients' visits and regular numbers of elective surgical procedures. The employment and income of healthcare staff continues to increase. On March 27, 2020, the USA federal government had announced a $2 trillion economic stabilization package entitled Coronavirus Aid, Relief, and Economic Security (CARES) Act to provide direct funding to the healthcare industry through a number of measures. Most significantly, the Act provides $100 billion to the Department of Health and Human Services (HHS) Public Health and Social Services Emergency Fund to be used for necessary expenses to reimburse for healthcare workers (Satiani et al., 2020). Australian general practices have undertaken major innovation and realignment response during the COVID-19 pandemic (Kippen et al., 2020). Telemedicine adoption has rapidly accelerated since the onset of the COVID-19 pandemic. Telemedicine provides increased access to medical care and helps to mitigate risk by conserving personal protective equipment and providing for social/physical distancing to continue to treat patients with a variety of chronic conditions. Therefore, utilizing telemedicine is ideal for ongoing safe treatment of patients, while continuing to promulgate responsible social and physical distancing in accordance with quarantine regulations in the hopes of slowing the spread of COVID-19 (Hare et al., 2020).

A heightened demand for medical specialties such as infectious disease, intensive care, and internal medicine were identified in our study. In our school, we expanded the admission number of the pulmonary and critical care medicine (PCCM) program and updated public health requirements during the training. Our employment guidance center carried out a recruitment training camp to provide systematic employment guidance throughout the pandemic. The hospital increased several job postings for teaching and research assistants for graduates to apply. Also, given that some students were unable to conduct their research and defend their thesis, we encouraged instructors to allow students to graduate based on their evaluation of these students' overall performance. For students who might be more susceptible to mental health problems, Department of Medical Graduates in our school has designated people to carefully monitor and help them by academic guidance and emotional health service.

Last but most importantly, medical graduates should be aware of the changed employment landscape and make suitable adaptations. During the pandemic period, students have free access to various academic resources, providing an early access for the completion of study design and graduation thesis. Our results showed that start date of job search was a good predictor of successful employment. The earlier a student started looking for jobs, the more prepared they may be, and the more likely they will successfully find employment. By July 2020, 27.8% of medical graduates reported having either just begun or not yet begun their job search.

## Conclusion

COVID-19 pandemic has a profound negative impact on the career-advancement and mental health of medical school graduates. Medical graduates who started early to find jobs and had high self-efficacy were more likely to get employed. The Chinese government has taken immediate and extraordinary measures to control the COVID-19 outbreak and brought the medical environment back to normal. It seems possible that the employment rate could increase in 2021, beyond what was experienced in 2020, with timely employment-promotion measures from government, schools, and medical students to improve the pandemic situation in China.

## Recommendations

In terms of employment statistics collection, the federal and local human resources should make flexible adjustments and delay the time frame of employment data statistics. Beijing Municipal Bureau of Human Resources and Social Security post-poned the employment agreement window from March 1 to December 31, 2020 (CCTV, 2020). Whether similar policies can be implemented on a broader scale needs to be carefully deliberated. Online platforms and facilities were promoted to connect supply and demand and engage online interviews, contract signing, and employment check-in (Ministry of Human Resources Social Security, 2020). It also extended the time that unemployed graduates could keep their documents and registered residence files in their schools to 2 years after graduation. Government should advocate telemedicine employment to medical graduates (who are young enough to be familiar with telemedicine operations) and adjust new insurance policies for telehealth program to make the service more accessible.

Medical schools should promote curriculums related to disease control such as PCCM program and public health. Online interview skill courses are needed as lower performance ratings and negative perceptions were more common in online interviews than in face-to-face interviews. Online career planning courses should be developed for graduates to receive virtual training in practical skills and social adaptability, to make the graduates more competitive for emerging jobs and shaping their self-efficacy. Medical schools should be aware of the impact of COVID-19 and provide psychological support and employment assistance. Schools need to be prepared for those who might be more susceptible to mental-health problems during a pandemic. Referrals for health services and additional academic support may be necessary to supplement their current studies and subsequent terms.

Medical graduates themselves should start early to find employment. Plus, multichannel of employments should be advocated. For example, telemedicine is now poised for rapid growth and recruitment of young doctors. Internet-based medical service platforms such as We Doctor, Spring Rain Doctor, etc., were developed and get rid of many conventional constraints upon its expansion. Online broadcasting channels, such as Douyin, Podcast and so on, were also effective tracks for self-employment.

## Data Availability Statement

The original contributions presented in the study are included in the article/supplementary material, further inquiries can be directed to the corresponding author.

## Ethics Statement

This research is approved by the Ethical Committee of Xiangya Hospital, CSU (Approval #202006069). This study did not include experiments on animal or human subjects. Consent was received from the participants to use the information anonymously for the purpose of this study.

## Author Contributions

Data collection was performed by XZ, XX, XL, and LZ. Data analysis was performed by XZ and MX. The first draft of the manuscript was written by MX. All authors contributed to the study conception and design, commented on previous versions of the manuscript, approved the final manuscript, and are accountable for all aspects of the work.

## Funding

This research was supported by the key project of graduate education teaching reform in Central South University (#2020JGA001), and degree and graduate education teaching reform research project in Hunan Province (#JG2018B015).

## Conflict of Interest

The authors declare that the research was conducted in the absence of any commercial or financial relationships that could be construed as a potential conflict of interest.

## Publisher's Note

All claims expressed in this article are solely those of the authors and do not necessarily represent those of their affiliated organizations, or those of the publisher, the editors and the reviewers. Any product that may be evaluated in this article, or claim that may be made by its manufacturer, is not guaranteed or endorsed by the publisher.
